# Non-invasive assessment of central venous pressure in heart failure: a systematic prospective comparison of echocardiography and Swan-Ganz catheter

**DOI:** 10.1007/s10554-020-01889-3

**Published:** 2020-05-22

**Authors:** Tobias Szymczyk, Odile Sauzet, Lech J. Paluszkiewicz, Angelika Costard-Jäckle, Max Potratz, Volker Rudolph, Jan F. Gummert, Henrik Fox

**Affiliations:** 1 Clinic for General and Interventional Cardiology/Angiology, Herz- und Diabeteszentrum NRW, Ruhr-Universität Bochum, Bad Oeynhausen, Germany; 2 Epidemiology and International Public Health, Bielefeld School of Public Health and Statistical Consulting Centre, Bielefeld University, Bielefeld, Germany; 3 Clinic for Thoracic and Cardiovascular Surgery, Herz- und Diabeteszentrum NRW, Ruhr-Universität Bochum, Georgstraße 11, 32545 Bad Oeynhausen, Germany; 4 Heart Failure Department, Herz- und Diabeteszentrum NRW, Ruhr-Universität Bochum, Bad Oeynhausen, Germany

**Keywords:** Heart failure, Inferior vena cava, Echocardiography, Central venous pressure, Right-heart catheterization

## Abstract

Assessing hemodynamics, especially central venous pressure (CVP), is essential in heart failure (HF). Right heart catheterization (RHC) is the gold-standard, but non-invasive methods are also needed. However, the role of 2-dimensional echocardiography (2DE) remains uncertain, and 3-dimensional echocardiography (3DE) is not always available. This study investigated standardized and breathing-corrected assessment of inferior vena cava (IVC) volume using echocardiography (2DE and 3DE) versus CVP determined invasively using RHC. Sixty consecutive HF patients were included (82% male, age 54 ± 11 years, New York Heart Association class 2.23 ± 0.8, ejection fraction 46 ± 18.4%, brain natriuretic peptide 696.93 ± 773.53 pg/mL). All patients underwent Swan-Ganz RHC followed by 2DE and 3DE, and IVC volume assessment. On 2DE, mean IVC size was 18.3 ± 5.5 mm and 13.8 ± 6 mm in the largest deflection and shortest distention, respectively. Mean CVP from RHC was 9.3 ± 5.3 mmHg. Neither 2DE nor 3DE showed acceptable correlation with invasively measured CVP; IVC volume acquisition showed optimal correlation with RHC CVP (0.64; 95% confidence interval 0.46–0.77), with better correlation when mitral valve early diastole E wave and right ventricular end-diastolic diameter were added. Using a CVP cut-point of 10 mmHg, receiver operating characteristic curve showed true positivity (specificity) of 0.90 and sensitivity of 62% for predicting CVP. A validation study confirmed these findings and verified the high predictive value of IVC volume assessment. Neither 2DE nor 3DE alone can reliably mirror CVP, but IVC volume acquisition using echocardiography allows non-invasive and adequate approximation of CVP. Correlation with invasively measured pressure was strongest when CVP is > 10 mmHg.

## Introduction

Heart failure (HF) prevalence is increasing, with a progressive disease course, poor prognosis and impaired quality of life [1]. Guiding therapy in individual HF patients remains challenging, and hemodynamics and filling pressures play a major role, particularly in predicting rehospitalization and mortality [2]. Assessing hemodynamic parameters, including central venous pressure (CVP), is essential in HF for both diagnostics and therapy guidance, but there is currently no non-invasive, quick, and easy-to-use technique for reliable measurement of CVP.

CVP has been shown to be an independent predictor of cardiac rehospitalization in patients with acute decompensated HF presenting to an emergency room [3]. Volume overload represents the leading cause of HF decompensation [4], but simple and reliable methods for determining volume overload in HF are lacking [5]. Established clinical appraisal of venous congestion, such as jugular vein distension is known to be unreliable and differs between observers [5, 6], although venous congestion has been demonstrated to be the leading predictor of organ injury and death [7].

Today, invasive assessment of CVP remains the only reliable approach to identify volume overload in HF, but the “gold-standard” Swan-Ganz catheter right heart catheterization (RHC) is costly, uncomfortable for patients, and associated with procedural risks such as pneumothorax, cardiac arrhythmia, bleeding, infection, nerve or vessel injury [8]. Therefore, there is a need for a non-invasive alternative but none is currently available. Most clinicians take the approach of estimating CVP using two-dimensional echocardiography (2DE) to estimate inferior vena cava (IVC) size in the subcostal view [9]. However, interobserver variability is high and agreement with invasive measurement is poor [10]. This is due to the often elliptical and irregular shape of the IVC and the variegating angle in which the ultrasound window depicts the vessel, making it impossible to capture the real dimension of the IVC with any two-dimensional approach [9]. It has been suggested that three-dimensional echocardiography (3DE) has the potential to capture CVP, but time consuming vessel area registration and complex calculations of a collapsibility and eccentricity index are needed to achieve reproducible and accurate measurements, making this approach impractical in daily clinical routine [9]. Cross-section measurements of IVC short and long axis were found to have potential for estimating CVP, but this was mainly limited to the ability to detect CVP of < 10 versus ≥ 10 mmHg [11].

Due to the high clinical impact and importance of accurate CVP measurement for determining prognosis, guiding HF therapy, preventing secondary organ damage, and evaluating suitability for heart transplantation, this study investigated the best possible approach for ultrasound-based non-invasive CVP measurements using echocardiography compared with RHC, including relevant confounders.

## Methods

### Study design

This prospective, single-center, observational study included consecutive HF patients from an academic medical center in Bad Oeynhausen, Germany (Herz- und Diabeteszentrum Nordrhein-Westfalen, Ruhr-University Bochum). The trial was registered at ClinicalTrials.gov (NCT03231774).

### Study population

Eligible patients were scheduled for routine HF check-up and underwent routinely planned echocardiography and RHC. Exclusion criteria were age < 18 years, chronic kidney disease requiring dialysis, and the presence of an implanted total artificial heart device.

### Right heart cardiac catheterization

Swan-Ganz RHC was performed in all study participants, immediately followed by echocardiography to ensure the same volume load for both examination methods. After setting-up routine monitoring (including ECG, non-invasive blood pressure and pulse oximetry), pulmonary artery catheterization (Edwards Lifesciences, Irvine, CA, USA) was performed by introduction into right internal jugular vein by experienced operators only. The Swan-Ganz pulmonary artery catheter was then advanced to first determine CVP, followed by other right heart pressure values.

### Two-dimensional echocardiography

Comprehensive transthoracic echocardiographic examinations were performed using Philips EPIQ 7 G ultrasound system (Philips Healthcare, Koninklijke Philips N.V., Amsterdam, The Netherlands) using variable-frequency 1.0 to 5 MHz sector transducers (Philips X5-1- transducer). All echocardiographic recording included assessment of both the left and right heart. Including established standard measures, we assessed left ventricular end-diastolic volume, left ventricular end-systolic volume and left ventricular ejection fraction, using standardized Simpson’s method in both four and two chamber view. Additional collected parameters contained left atrial volume, pulsed Doppler transmitral flow profiles, pulsed wave Doppler placed in the left ventricular outflow tract, and tissue Doppler Imaging on the mitral annulus. Right heart function was assessed using right ventricular end-diastolic volume, tricuspid annular plane systolic excursion, and fractional area change of the right ventricle. The velocity flow profile in hepatic veins was measured by pulsed wave Doppler from the subcostal view and the pulsed wave Doppler flow profile of the upper right pulmonary vein was recorded. Strain analysis was performed and the Myocardial Performance Index (Tei-Index) was calculated. The size of the IVC was determined with the patient in the supine position using 2DE images from the subxyphoidal view. The breathing-corrected diameter was measured perpendicular to the long-axis of the IVC, proximal to the junction of the hepatic veins, 2.0 cm proximal to the ostium of the right atrium, assessing IVC volume as largest deflection and shortest distention.

### Three-dimensional echocardiography

**Fig. 1:**
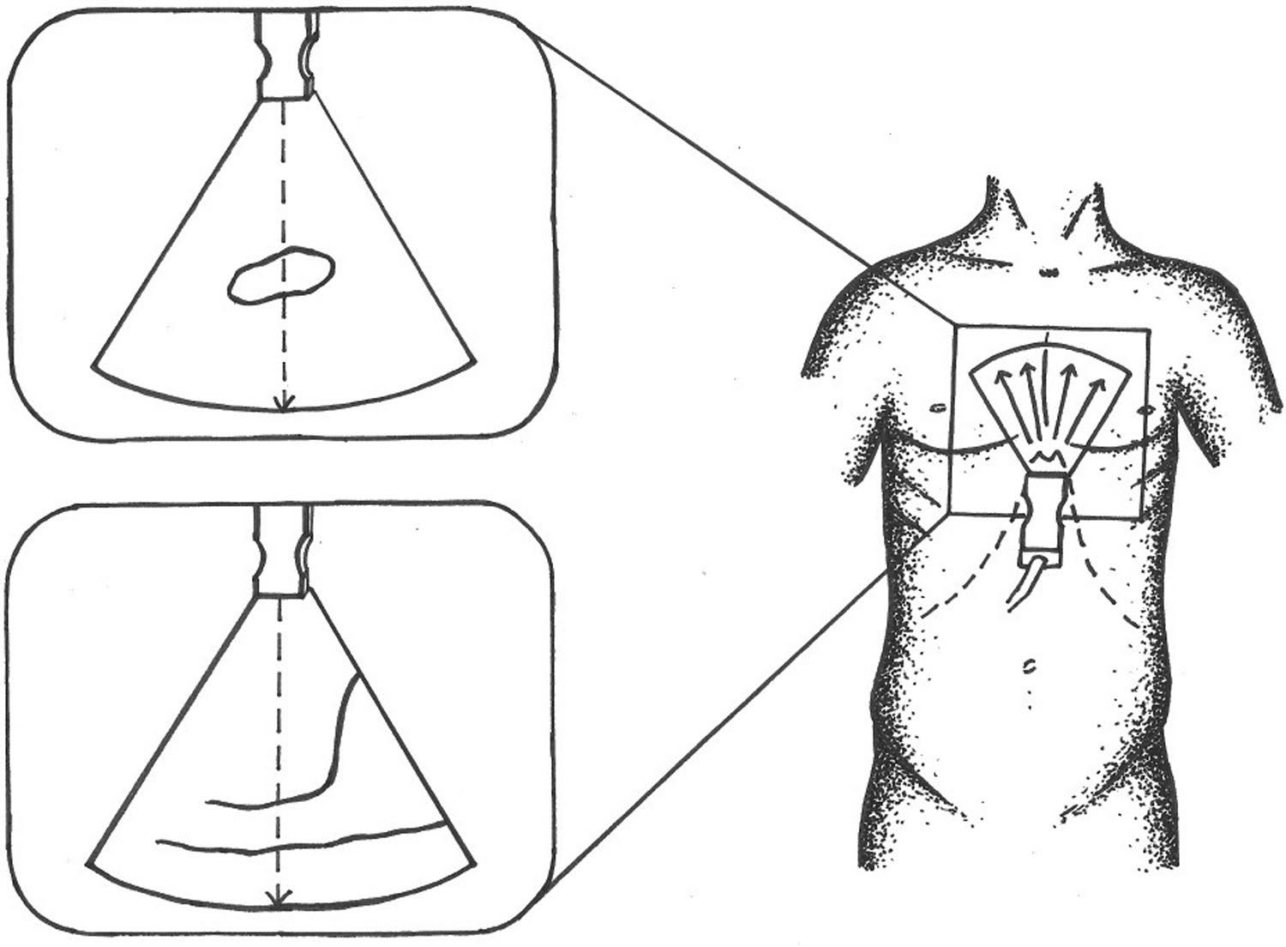
Schematic visualization of echocardiography technique used in this study. This figure illustrates the standardized consistent measurement of inferior vena cava (IVC) and vena cava inferior volume, defined as the product of largest deflection and shortest distention, used in this study. Transducer placed in predefined standardized subxyphoidal position. Patient in supine position and IVC diameters are measured, breathing corrected, perpendicular to IVC long-axis, proximal to the junction of the hepatic veins, 2.0 cm proximal to the ostium of the right atrium, assessing IVC volume as largest deflection and shortest distention

Single full-volume 3DE loop data were acquired using a subxyphoidal view with the patient in a supine position. 3DE full-volume images of standardized breathing-corrected sounding IVC were recorded, placed approximately 2.0 cm (0.79 inches) proximal to the ostium of the right atrium and perpendicular to the long-axis reference of the IVC. Digital cross-sectional image reconstruction was done using available software (Philips QLAB 10, QLAB Ultrasound Cardiac Analysis, Koninklijke Philips N.V., Amsterdam, The Netherlands) for cardiac 3D quantification. IVC cross-sectional images were scaled into a vertical diameter and in a second diameter perpendicular to the vertical diameter. All measurements were compiled for their largest and smallest deflection. In addition, IVC area was quantified in the cross-sectional images to picture frequent irregular IVC morphology. Schematic presentation of exemplary echocardiographic examination with ultrasound images in cross-sectional and longitudinal-sectional view of IVC is shown in Fig. 1.

### Statistical analysis

Firstly, the correlation between IVC measures and CVP was estimated. Then a first linear regression model was selected that could best predict CVP using IVC measures and demographic values (Model 1). 3DE was not available in all patients due to poor image quality; instead, IVC volume was assessed using orthogonal transducer locations. In the second phase, a large number of non-invasive routinely collected measures with the potential to improve the above prediction model were considered (Model 2). All measures for which data were available for at least 50/60 were considered to ensure that there were enough observations to imply a linear regression model. Next, stepwise selection-based analysis was performed, using the sufficient sample size of at least 50 observations and based on Akaike information criterion (AIC) and R-squared (R^2^) values. The models were always compared with the dataset including all complete cases for the comprehensive set of all variables under consideration. Variables for which there was a smaller dataset were considered for sensitivity analyses. The predictive model was then validated by randomly splitting the dataset into two equal parts, one to obtain regression parameters for the model and one to test the predictive values of the final model.

The predictive values of Model 2 were tested by considering the distribution of the difference between predicted values and observed values, and by using a CVP diagnostic threshold of 10 mmHg and obtaining the area under the receiver operating characteristic (ROC) curve. Analyses were performed using Stata 14 (StataCorp. 2015. Stata Statistical Software: Release 14. College Station, TX: StataCorp LP), plots were realized using Sigma- Plot 12.0 by Systat Software Inc.

### Reproducibility

Echocardiography was performed by independent, blinded, experienced sonographers to exclude any examiner-dependent confounding. Moreover, both examiner groups for either echocardiography, as well as for Swan-Ganz catherization, were blinded from the results of the other group, to avoid influencing of any kind between the two methods. All software-based measurements were measured more than three times for each case, and those with if a deviation of > 5% were discarded.

## Results

A total of 60 patients were screened and fulfilled inclusion criteria for study participation (Table 1). All patients underwent complete clinical assessment including Swan-Ganz RHC followed by immediate 2DE and 3DE (Philips EPIQ 7G) examination including inferior vena cava (Fig. 1). Echocardiographic characteristics and catheterization data are presented in Table 2. All patients were assessed using 2DE, but 3DE data from three patients had to be excluded for poor image quality. Our inter- and intra-observer variability with 8% and below 5%, was comparable to similar studies in the literature. The results of the correlation analysis are presented in Table 3, including sample size for each variable.

**Table 1:** Baseline characteristics of the study population

	Patients (n = 60)
Demographic data	
Age (years)	54 ± 11
Male, n (%)	49 (82)
Height (cm)	177 ± 8
Weight (kg)	87 ± 16
Body mass index (kg/m^2^)	28 ± 5
Body surface area (m^2^)	2.03 ± 0.2
NYHA class	2.23 ± 0.8
Heart rhythm	
Heart rate (beats/min)	74 ± 14
Sinus rhythm, n (%)	49 (82)
Pacemaker mediate rhythm, n (%)	19 (32)
Atrial fibrillation, n (%)	8 (13)
Blood values	
Sodium (mmol/L)	138 ± 2.8
Potassium (mmol/L)	4 ± 0.4
Creatinine (mg/dL)	1.7 ± 1.5
Glomerular filtration rate (mL/min)	56 ± 23
Hematocrit (%)	39 ± 5
Hemoglobin (g/dL)	13 ± 1.8
Brain natriuretic peptide (ng/L)	696.93 ± 773.53
Underlying disease, n (%)	
Ischemic etiology	17 (28)
Dilated cardiomyopathy	27 (45)
Other cardiomyopathy	16 (27)
Other clinical characteristics, n (%)	
Diabetes	13 (22)
LVAD	7 (12)
Coronary artery disease	9 (15)

Values are mean values ± standard deviation, or number of patients (%)

*LVAD* left ventricular assist device, *NYHA* New York Heart Association

**Table 2:** Echocardiographic and right heart catherization parameters

	Values
Echocardiography parameters	
LVEF (%)	46 ± 18.4
LVEDD (mm)	62 ± 14
LVESD (Mm)	52 ± 17
LVEDV (mL) strain	207 ± 120
LVESV (mL) strain	128 ± 96
LAVI (mL/m^2^)	60 ± 28
E (cm/s)	68 ± 29
E/E′ ratio	11.9 ± 7
TAPSE (mm)	17.8 ± 4
RV FAC (%)	30.3 ± 12.8
RVEDD (mm)	42 ± 9.4
Moderate or severe TR, n (%)	19 (32)
Vena cava inferior parameters in echocardiography	
IVC 2DE maximal deflection (mm)	18.3 ± 5.5
IVC 2DE minimal deflection (mm)	13.8 ± 6
IVC 3DE maximal deflection vertical axis (mm)	16.9 ± 7.3
IVC 3DE maximal deflection perpendicular axis (mm)	22 ± 8.6
IVC 3DE minimal deflection vertical axis (mm)	13.8 ± 7.7
IVC 3DE minimal deflection perpendicular axis (mm)	16.7 ± 8
IVC 3DE area maximal deflection (cm^2^)	3.58 ± 2.54
IVC 3DE area minimal deflection (cm^2^)	2.49 ± 2.33
Invasive right heart catheterization parameters	
CVP	9.3 ± 5.3
Mean RVP (mmHg)	17.6 ± 8.7
Mean PAP (mmHg)	24.7 ± 10.9
PCWP (mmHg)	15.9 ± 8
Cardiac index (L/min/m^2^)	2.1 ± 0.7

Values are mean values ± standard deviation, or number of patients (%)

*2DE* two-dimensional echocardiography, *3DE* three-dimensional echocardiography, *CVP* central venous pressure, *E* early wave, *E/E′* early wave doppler/early wave tissue doppler, *IVC* inferior vena cava, *LAVI* left atrial volume index, *LVEDD* left ventricular end-diastolic diameter, *LVEDV* left ventricular end-diastolic volume, *LVEF* left ventricular ejection fraction, *LVESD* left ventricular end-systolic diameter, *LVESV* left ventricular end-systolic volume, *PAP* pulmonary artery pressure, PCWP post-capillary wedge pressure, *RVEDD* right ventricular end-diastolic diameter, *RV FAC* right ventricular fractional area change, *RVP* right ventricular pressure, *TAPSE* tricuspid annular plane systolic excursion, *TR* tricuspid regurgitation

**Table 3:** Correlation analyses

Measures	Correlation with CVP R^2^ (95% CI)
IVC 2DE maximal deflection (mm)	0.58 (0.38, 0.73)
IVC 2DE minimal deflection (mm)	0.57 (0.37, 0.72)
IVC volume (mm^2^)	0.64 (0.46, 0.77)
IVC 3DE maximal deflection vertical axis (mm)	0.32 (0.03, 0.55)
IVC 3DE maximal deflection perpendicular axis (mm)	0.51 (0.26, 0.67)
IVC 3DE minimal deflection vertical axis (mm)	0.32 (0.04, 0.56)
IVC 3DE minimal deflection perpendicular axis (mm)	0.50 (0.26, 0.69)
IVC 3DE area maximal deflection (cm^2^)	0.49 (0.24, 0.68)
IVC 3DE area minimal deflection (cm^2^)	0.46 (0.19, 0.67)

*2DE* two-dimensional echocardiography, *3DE* three-dimensional echocardiography, *CI* confidence interval, *IVC* inferior vena cava, *IVC* volume, defined as the product of largest deflection and shortest distention

2DE CVP measurements did not show satisfactory correlation for CVP alone, but breathing-corrected IVC volume assessment showed some correlation with invasively measured CVP (R^2^ = 0.64). When adding supplementary patient characteristics to the predictive statistical model (e.g. weight, height and body mass index), the R^2^ value reached 0.47. Other 3DE and 2DE measurements were unsatisfactory, mainly due to limited image quality, reaching correlations with R^2^ values of around 0.5 only.

**Fig. 2:**
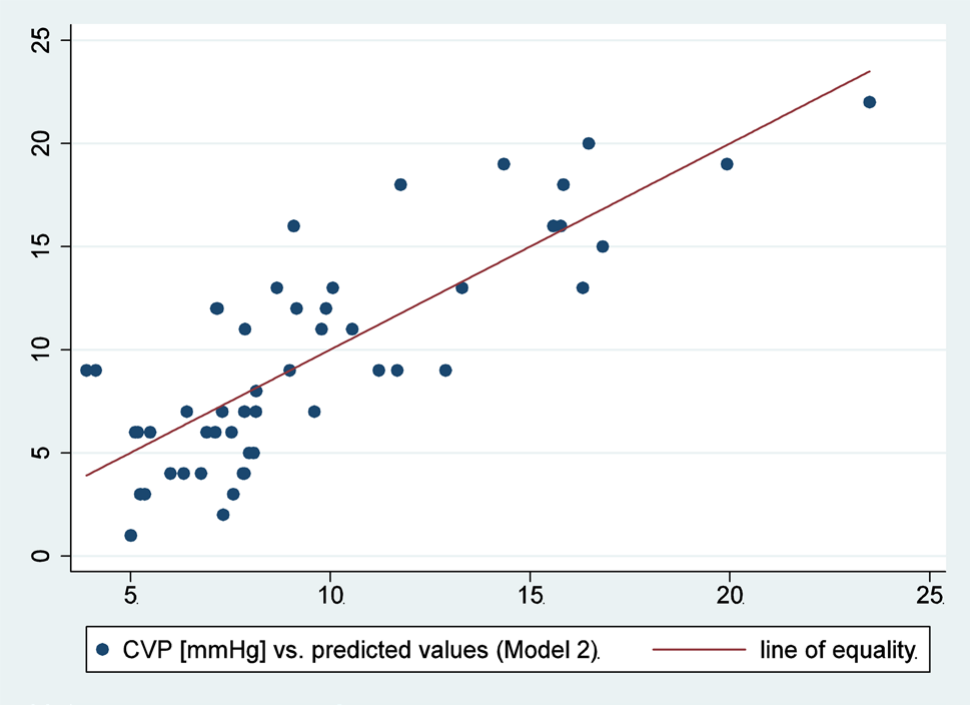
Correlation of central venous pressure (CVP) correlation with prediction model. Scatter plot of observed CVP values against predicted values from statistical model 2

Results from the linear model for IVC volume are provided in Table 4. Our model enhancement practice for a fully-optimized prediction model resulted in addition of the established variables mitral valve early diastole E wave (MVED) and right ventricular end-diastolic diameter (RVEDD) to the first model. The new model had a R^2 ^squared value of 0.66. The scatter plot of this linear prediction and its observed values of CVP are presented in Fig. 2, and show a regular distribution of the reading points around the correlation line in equality, suggesting good predictive value. Sensitivity analyses disclosed a CVP threshold of 10 mmHg to be most exact, which above all is clinically the utmost used value. When using a threshold for predicting CVP of 8 mmHg, sensitivity was 86%, specificity 74%, and a threshold for predicting CVP of 12 mmHg, sensitivity was 48%, specificity 97%.

**Table 4:** Variables and regression coefficients from statistical model 1 and 2

	Model 1 (n = 60)	Model 2 (n = 52)
	Coefficient (SE)	Coefficient (SE)
BMI (kg/m^2^)	− 2.68 (1.26)	− 2.30 (1.15)
Weight (kg)	0.92 (0.40)	0.79 (0.37)
Height (cm)	− 0.85 (0.40)	− 0.78 (0.35)
IVC volume (mm^2^)	0.012 (0.003)	0.01 (0.002)
Mitral valve early diastole E wave (cm/s)		0.04 (0.02)
Right ventricular end-diastolic diameter (mm)		0.14 (0.06)
R^2^	0.47	0.66

*BMI* body mass index, *E* early wave, *R*^*2*^ coefficient of determination, *SE* standard error

**Fig. 3:**
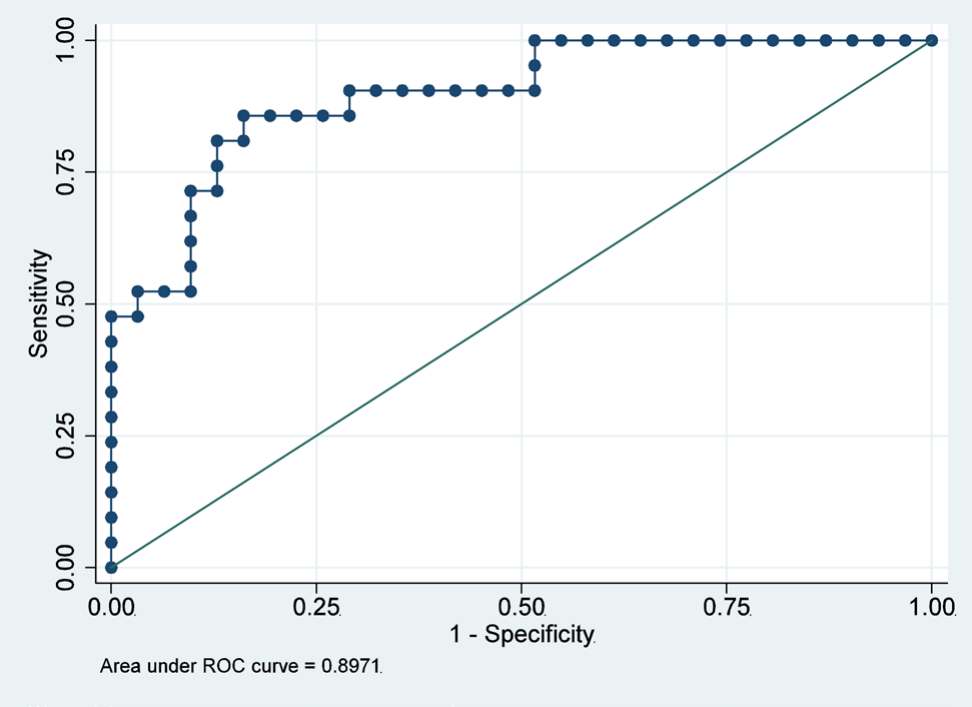
Sensitivity of statistical model 2 for central venous pressure (CVP) prediction. Receiver operating characteristic (ROC) curve for the predicted values from statistical model 2 and a CVP cut-point of 10 mmHg, under consideration of mitral valve early diastole E wave and right ventricular end-diastolic diameter. Area under the ROC curve (AUC): 0.90 (95% confidence interval 0.81–0.98)

Clinically most useful CVP cut-point of 10 mmHg resulted in an area under the ROC curve (Fig. 3) of 0.90 (95% confidence interval [CI] 0.81–0.98), with specificity of 90% and sensitivity of 62%. The sensitivity analysis showed that this prediction model could not be improved by any other additional variables. The positive predictive value is 81% and the negative predictive value is 78%.

**Fig. 4:**
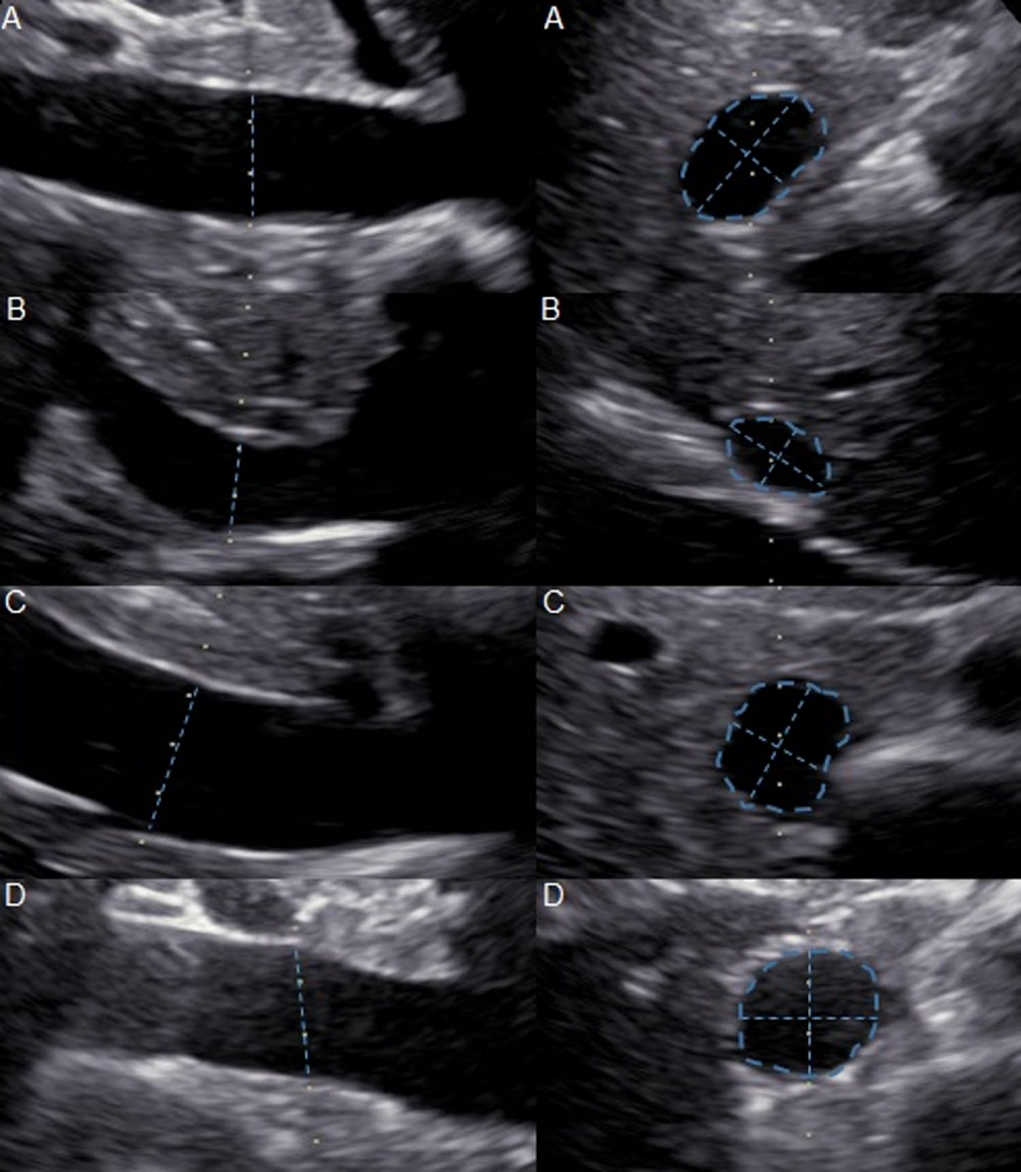
Ultrasound varieties of vena in three-dimensional view illustrating the wide differences of two-dimensional presentations. **a** Vena cava in an ellipsoid shape. **b** Vena cava in an egg-like shape. **c** Vena cava in a kidney-like shape. **d** Vena cava in round, ball-like shape

The broad variety of vena cava shapes identified are depicted in Fig. 4, illustrating the challenge of standardized assessment of vena cava size.

**Fig. 5:**
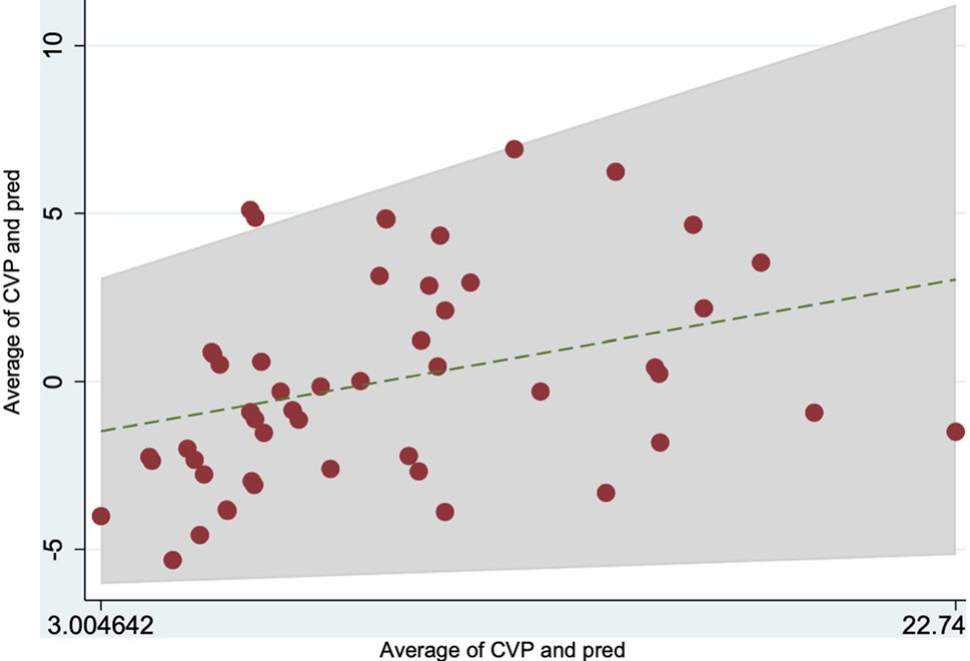
Bland–Altman plot for central venous pressure

**Fig. 6:**
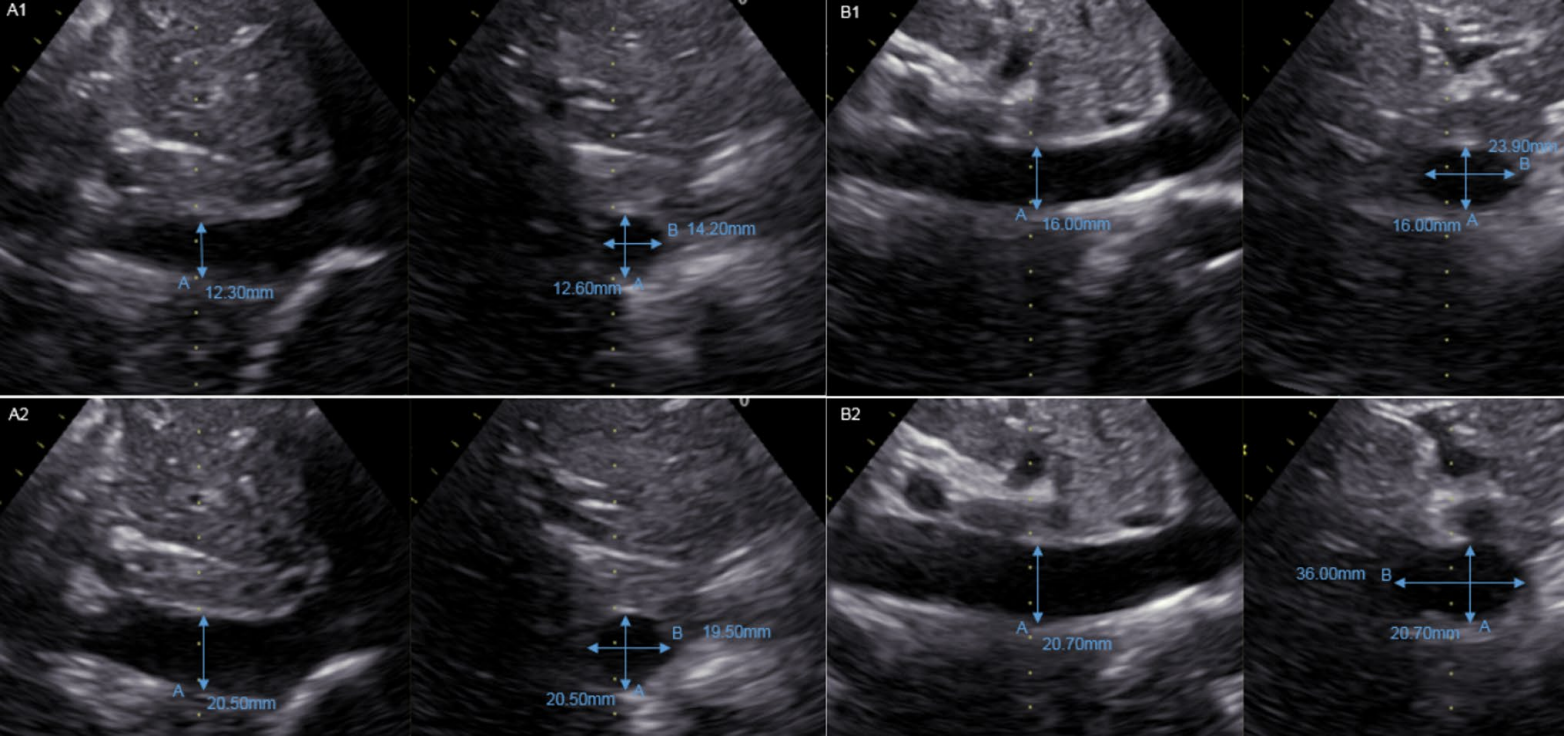
Inferior vena cava volume calculation—Inferior vena cava images including vectors. **a1** Example of a smaller roundish-shaped inferior vena cava with orthogonal vectors for volume. **a2** Example of a congested roundish-shaped inferior vena cava with orthogonal vectors for volume calculation. **b1** Example of a decongested ellipsoid-shaped inferior vena cava with orthogonal vectors for volume calculation. **b2** Example of a congested ellipsoid-shaped inferior vena cava with orthogonal vectors for volume calculation

### Validation

The validation study was performed to confirm the findings using the identified variables from Model 2; it involved parameters estimated from 26 randomly selected observations and we obtained the predictive values of that model with the remaining observations. Using the 10 mmHg CVP cut-point, we achieved an area under the ROC curve of 0.83 (95% CI 0.67–0.99), with specificity of 50% and sensitivity of 93%.

## Discussion

This study identified a reliable 90% predictive non-invasive approach to approximate CVP in patients with HF using IVC volume measurement based on standardized, breathing-corrected echocardiography data. Findings were compared against invasive, gold-standard Swan-Ganz RHC, and the statistical correlation model was improved when routinely collected echocardiography parameters (MVED and RVEDD) were taken into account (area under ROC of 0.90). This study also confirmed the poor correlation between traditional 2DE to predict CVP, but 2DE remains part of a broad standard of care in many echocardiography laboratories worldwide [10]. Thus, our findings document an easy-to-use and easy-to-implement approach to enhance CVP prediction that shows good correlation with invasive measurement. We were also able to validate an easy-to-use prediction model for CVP in HF that had excellent specificity and satisfactory sensitivity to predict CVP at a 10-mmHg threshold and can easily be incorporated into daily clinical practice (Figs. 5, 6).

Obtaining CVP is an essential component of HF treatment and CVP has been shown to guide prognosis [3]. Despite the need for a reliable, non-invasive method for estimating CVP, there are several challenges to identifying a feasible, consistent and practical approach, as discussed previously [5]. There are a number of factors that influence CVP, making a simple estimation from a single freeze ultrasound image impossible [5]. Therefore, techniques such as having the patient sniff have been used to acquire additional information for better estimation of CVP [12]. However, these approaches are limited by interobserver variability in assessing IVC collapse and venous compliance [10].

Similar to our results, Huguet et al. reported discrepancy for 2DE measurements of CVP, showing systematic underestimation, mostly for 2DE foreshortening artifacts [9]. Given the clinical implications of such measurements in guiding medical strategy in HF decompensation or cardiogenic shock, the discrepancies between 2DE estimations and actual CVP values are concerning [13]. Thus, a more accurate noninvasive approach for CVP assessment is required [14].

3DE is widely available, simple, can be performed at the bedside and is already part of daily practice, making it a better choice for determining comprehensive IVC geometry [11]. Respiratory position influences IVC geometry [9], which is why our standardized study measurements were reported as breathing-corrected values; in addition, we did not require an additional “sniff” test. Moreover, in contrast to other studies, we investigated an unselected, all-comers HF population, making our findings potentially applicable to a broad group of patients.

Huguet et al. reported significant correlation of IVC area in three-dimensional images with CVP [9]. In contrast, we found no good correlation of IVC area in cross-sectional images with invasively-measured CVP in our HF study population. Our statistical predictive model allows the best CVP approximation reported in literature yet. We used a novel approach to increase correlation, and ours is the first study to include left ventricular function parameters (MVED), along with the appropriate equipment and well-trained sonographers. This makes our approach not only reliable but also easily performable in a routine clinical setting.

With our study findings we encourage clinicians to attempt echocardiography based CVP approximations as a none-invasive alternative to Swan-Ganz catherization. Through implementation of our standardized, breathing corrected, orthogonal vector IVC volume assessment reliable prediction of a CVP > 10 mmHg is possible, which is of explicit clinical interest. When additional adding routinely obtained parameters of RVEDD and MVED we have been able to provide strongest correlation for a CVP above 10 mmHg. Our results suggest that echocardiography based IVC volume assessment may represent a feasible and easily applicable method for volume status evaluation in HF patients, with the potential to avoid risks of invasive catherization.

Larger studies in wider patient groups are needed to better determine the potential implications of our findings in clinical decision making and for guiding medical therapy in HF. 3DE IVC assessment may represent a more feasible and easily applicable method for evaluating volume status and measuring CVP in HF, eliminating the risks associated with RHC and preventing misinterpretation when 2DE estimates of IVC are used. However, 3DE is expensive and not widely available, making our simple method of IVC volume assessment relatively attractive. Additional calculations are necessary to determine the best predictive value of our findings. Ease of use of these techniques could be improved by incorporation of the underlying calculations into echocardiography software.

### Limitations

This study included a relatively small sample size, the majority of whom were male, and HF severity was moderate (mean NYHA class 2.23). Therefore, the findings can only be generalized to populations with similar characteristics to those in our study. The image quality of echocardiography measurements (both 2DE and 3DE) is limited in obese patients with HF, those who have had cardiac surgery and in patients with left ventricular assist devices, meaning that use of echocardiography to assess hemodynamics is not applicable in every patient. In addition, other comorbidities, such as chronic lung diseases, can also worsen image quality. Finally, our prediction model needs to be tested with data from other settings and centers and, in particular, its robustness to measurement errors needs further evaluation.

## Conclusion

2DE grading of IVC alone provided no reliable predictive value for CVP approximation compared with Swan-Ganz gold-standard of invasively measured CVP, limiting the role for this commonly-used technique in clinical decision making and medical therapy guidance. In contrast, 3DE facilitated identification not only of IVC size but also shape, allowing much better and more accurate CVP estimation. Thus, standardized IVC volume assessment imaging provided good information to allow prediction of CVP > 10 mmHg when the routinely obtained echocardiography parameters RVEDD and MVED were added (area under the curve 0.90) and correlation with invasively measured pressure was strongest when CVP is > 10 mmHg. Our results suggest that IVC volume assessment may represent a more feasible and easily applicable method for volume status evaluation and CVP measurement in HF, allowing the risks of RHC and misinterpretation based on 2DE data to be avoided.
